# *catena*-Poly[silver(I)-bis­[μ-bis­(diphenyl­phosphino)methane-κ^2^ *P*:*P*′]-μ-thio­cyanato-κ^2^ *S*:*S*-silver(I)-μ-thio­cyanato-κ^2^ *S*:*N*]

**DOI:** 10.1107/S1600536810035622

**Published:** 2010-09-11

**Authors:** Li-Li Song, Li-Na Cui, Qiong-Hua Jin, Cun-Lin Zhang

**Affiliations:** a Department of Chemistry, Capital Normal University, Beijing 100048, People’s Republic of China; b Beijing Key Laboratory for Terahertz Spectroscopy and Imaging, Key Laboratory of Terahertz Optoelectronics, Ministry of Education, Capital Normal University, Beijing 100048, People’s Republic of China

## Abstract

The title compound, [Ag(NCS)(C_25_H_22_P_2_)]_*n*_, contains two Ag^+^ ions, two thio­cyanate ions and two bis­(diphenyl­phosphino)methane (dppm) ligands in the asymmetric unit. One of the thiocyanate ions bridges the two Ag^+^ ions in a μ_2_-mode from its S atom and the two dppm ligands bridge the silver ions in a μ_1_,μ_1_ mode. The remaining SCN^−^ ion bridges the binuclear units *via* its N and S atoms, generating a one-dimensional polymer propagating in [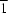
01]: the resulting AgP_2_SN and AgP_2_S_2_ coordination geometries could be described as distorted tetra­hedral.

## Related literature

For general background to silver(I) complexes, see: Awaleh *et al.* (2007 ▶); Liu *et al.* (2008 ▶). For silver(I) complexes containing phosphine ligands and coordinated anions, see: Jin, Song *et al.* (2010 ▶); Jin, Hu *et al.* (2010 ▶). For related structures, see: Jin *et al.* (2008 ▶); Cingolani *et al.* (2005 ▶).
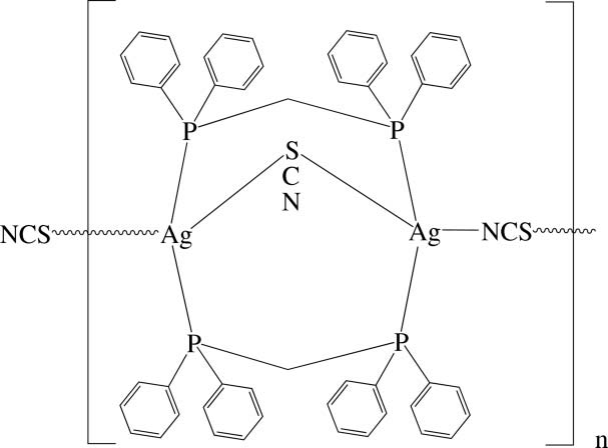


## Experimental

### 

#### Crystal data

[Ag(NCS)(C_25_H_22_P_2_)]
*M* *_r_* = 550.32
Monoclinic, 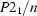

*a* = 13.0712 (14) Å
*b* = 23.080 (2) Å
*c* = 15.6340 (16) Å
β = 93.470 (1)°
*V* = 4708.0 (8) Å^3^
*Z* = 8
Mo *K*α radiation
μ = 1.10 mm^−1^
*T* = 298 K
0.43 × 0.34 × 0.32 mm

#### Data collection

Bruker SMART CCD area-detector diffractometer
Absorption correction: multi-scan (*SADABS*; Bruker, 2007 ▶) *T*_min_ = 0.650, *T*_max_ = 0.721
23675 measured reflections
8306 independent reflections
4539 reflections with *I* > 2σ(*I*)
*R*_int_ = 0.056

#### Refinement

*R*[*F* ^2^ > 2σ(*F* ^2^)] = 0.059
*wR*(*F* ^2^) = 0.168
*S* = 1.02
8306 reflections
559 parameters
1 restraint
H-atom parameters constrained
Δρ_max_ = 1.34 e Å^−3^
Δρ_min_ = −1.31 e Å^−3^

### 

Data collection: *SMART* (Bruker, 2007 ▶); cell refinement: *SAINT-Plus* (Bruker, 2007 ▶); data reduction: *SAINT-Plus*; program(s) used to solve structure: *SHELXS97* (Sheldrick, 2008 ▶); program(s) used to refine structure: *SHELXL97* (Sheldrick, 2008 ▶); molecular graphics: *SHELXTL* (Sheldrick, 2008 ▶); software used to prepare material for publication: *SHELXTL*.

## Supplementary Material

Crystal structure: contains datablocks global, I. DOI: 10.1107/S1600536810035622/hb5620sup1.cif

Structure factors: contains datablocks I. DOI: 10.1107/S1600536810035622/hb5620Isup2.hkl

Additional supplementary materials:  crystallographic information; 3D view; checkCIF report

## Figures and Tables

**Table 1:** Selected bond lengths (Å)

Ag1—P1	2.450 (2)
Ag1—P3	2.451 (2)
Ag1—S1	2.670 (3)
Ag1—S2	2.768 (3)
Ag2—N2^i^	2.429 (9)
Ag2—P2	2.497 (2)
Ag2—P4	2.498 (2)
Ag2—S1	2.668 (2)

Symmetry code: (i) 
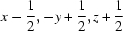
.

**Fig. 1.**
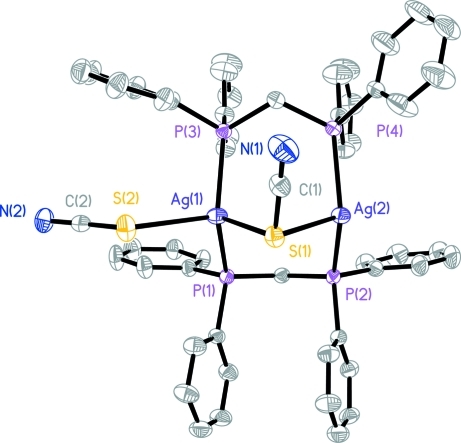
Perspective view of a basic unit of the title complex. Hydrogen atoms are omitted for clarity.

**Fig. 2.**
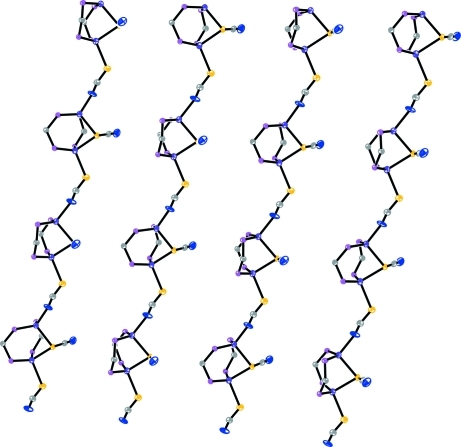
Perspective view of a one-dimensional polymer of the title complex.

## supplementary crystallographic information

### Comment

Many research efforts have been devoted to the silver(I) complexes due to their fascinating structures and potential applications in the field of photo-sensitizer, semi-conducting or catalytic devices (Awaleh *et al.*, 2007, Liu *et al.*, 2008). Recently, we have found some silver(I) complexes containing phosphine ligands and coordinated anions (Jin, Song *et al.*, 2010, Jin, Hu *et al.*, 2010). Continuing these efforts, we obtain a new one-dimensional polymer [Ag_2_(dppm)_2_(SCN)_2_] by using AgSCN, dppm and 1,10-Phenanthroline(phen).

The crystal structural analysis shows that SCN^-^ bridges the [Ag_2_(dppm)_2_(SCN)] binuclear units to give rise to a one-dimensional polymer. In the binclear unit, two dppm ligands lock two silver atoms to form a circular dimer, where S atom of SCN^-^ behaving as bridging ligand links two silver atoms. Two silver atoms are four-coordinated. Ag1 is coordinated by two P-atoms from two dppm ligands, two S atoms from two thiocyanide anions while Ag2 is coordinated by two P-atoms from two dppm ligands, one S-atom and one N-atom from two thiocyanide anions. In the title compound, the P—Ag1—S angles are in the range 104.55 (9)–108.67 (8)°, and the P—Ag1—P angle is 127.18 (8)°, S—Ag1—S is 99.11 (8)°; while N—Ag2—P are in the range 101.4 (2)–113.2 (2)°, P—Ag2—P is 126.43 (8)°, N—Ag2—S are in the range 101.5 (3)–106.66 (8)°. This confirms the distored tetrahedral environment around two silver (I) atoms. The distance of the two silver atoms (3.484 Å) is 0.048 Å longer than the sum of the covalent radii(3.44 Å), which indicates that there exists weak Ag···Ag interaction. The bond lengths of S1—Ag1 and S1—Ag2 are 2.670 and 2.668 Å, respectively.The angles of N1—C1—S1 and N2—C2—S2 are 179.55 and 165.33°, respectively. The angle of Ag1—S1—Ag2 are 10.92° shorter than that reported for [Ag_2_(dpam)_2_(SCN)_2_](92.39°) (Cingolani *et al.*, 2005).

The similar compound [Ag_4_(SCN)_4_(dppm)_2_] (Jin *et al.*,2008) is prepared by the similar reaction by using quinoline in place of phen. Though both quinoline and phen don't take part in coordination, this two ligands effect the final structures of the products. This confirms again that different nitrogen heterocyclic ligands lead to different structures due to the subtle interation of the nitrogen heterocyclic ligands with silver ions. (Jin, Hu *et al.*, 2010).

### Experimental

A mixture of AgSCN (0.0332 g, 0.2 mmol), 1,10-Phenanthroline (0.0792, 0.4 mmol) and bis(diphenylphosphino)methane (0.1532 g, 0.4 mmol) in the molar ratio 1:2:2 in CH_3_OH/CH_2_Cl_2_ was stirred for 5 h at ambient temperature. After filtration, the filtrate was allowed to stand still. Slow evaportation of the solvent yielded colourless blocks of (I). Analysis found(percentage): C 56.70, H 4.00, N 2.54; calculated: C 56.21, H 3.72, N 2.24.

### Refinement

All hydrogen atoms were located in the calculated sites and included in the final refinement in the riding model approximation with displacement parameters derived from the parent atoms to which they were bonded.

### Crystal data

**Table:**

[Ag(NCS)(C_25_H_22_P_2_)]	*F*(000) = 2224
*M**_r_* = 550.32	*D*_x_ = 1.553 Mg m^−^^3^*D*_m_ = 1.553 Mg m^−^^3^*D*_m_ measured by not measured
Monoclinic, *P*2_1_/*n*	Mo *K*α radiation, λ = 0.71073 Å
Hall symbol: -P 2yn	Cell parameters from 4221 reflections
*a* = 13.0712 (14) Å	θ = 2.3–23.1°
*b* = 23.080 (2) Å	µ = 1.10 mm^−^^1^
*c* = 15.6340 (16) Å	*T* = 298 K
β = 93.470 (1)°	Block, colourless
*V* = 4708.0 (8) Å^3^	0.43 × 0.34 × 0.32 mm
*Z* = 8	

### Data collection

**Table:**

Bruker SMART CCD area-detector diffractometer	8306 independent reflections
Radiation source: fine-focus sealed tube	4539 reflections with *I* > 2σ(*I*)
graphite	*R*_int_ = 0.056
phi and ω scans	θ_max_ = 25.0°, θ_min_ = 1.6°
Absorption correction: multi-scan (*SADABS*; Bruker, 2007)	*h* = −15→15
*T*_min_ = 0.650, *T*_max_ = 0.721	*k* = −27→22
23675 measured reflections	*l* = −15→18

### Refinement

**Table:**

Refinement on *F*^2^	Primary atom site location: structure-invariant direct methods
Least-squares matrix: full	Secondary atom site location: difference Fourier map
*R*[*F*^2^ > 2σ(*F*^2^)] = 0.059	Hydrogen site location: inferred from neighbouring sites
*wR*(*F*^2^) = 0.168	H-atom parameters constrained
*S* = 1.02	*w* = 1/[σ^2^(*F*_o_^2^) + (0.0414*P*)^2^ + 37.553*P*] where *P* = (*F*_o_^2^ + 2*F*_c_^2^)/3
8306 reflections	(Δ/σ)_max_ = 0.001
559 parameters	Δρ_max_ = 1.34 e Å^−^^3^
1 restraint	Δρ_min_ = −1.31 e Å^−^^3^

### Special details

**Table:**

Geometry. All e.s.d.'s (except the e.s.d. in the dihedral angle between two l.s. planes) are estimated using the full covariance matrix. The cell e.s.d.'s are taken into account individually in the estimation of e.s.d.'s in distances, angles and torsion angles; correlations between e.s.d.'s in cell parameters are only used when they are defined by crystal symmetry. An approximate (isotropic) treatment of cell e.s.d.'s is used for estimating e.s.d.'s involving l.s. planes.
Refinement. Refinement of *F*^2^ against ALL reflections. The weighted *R*-factor *wR* and goodness of fit *S* are based on *F*^2^, conventional *R*-factors *R* are based on *F*, with *F* set to zero for negative *F*^2^. The threshold expression of *F*^2^ > σ(*F*^2^) is used only for calculating *R*-factors(gt) *etc*. and is not relevant to the choice of reflections for refinement. *R*-factors based on *F*^2^ are statistically about twice as large as those based on *F*, and *R*- factors based on ALL data will be even larger.

### Fractional atomic coordinates and isotropic or equivalent isotropic displacement parameters (Å^2^)

**Table:**

	*x*	*y*	*z*	*U*_iso_*/*U*_eq_	
Ag1	0.67704 (5)	0.24042 (3)	0.55934 (5)	0.0460 (2)	
Ag2	0.49477 (5)	0.24103 (3)	0.71296 (4)	0.0422 (2)	
N1	0.7422 (7)	0.1363 (5)	0.7966 (7)	0.090 (3)	
N2	0.9491 (8)	0.2412 (4)	0.3589 (6)	0.084 (3)	
P1	0.60071 (16)	0.33099 (9)	0.50436 (14)	0.0342 (5)	
P2	0.43500 (16)	0.33225 (9)	0.64005 (14)	0.0343 (5)	
P3	0.61866 (16)	0.14163 (9)	0.52595 (14)	0.0336 (5)	
P4	0.45647 (17)	0.14001 (9)	0.66334 (15)	0.0379 (5)	
S1	0.69887 (17)	0.24681 (11)	0.73000 (16)	0.0488 (6)	
S2	0.8817 (2)	0.24072 (14)	0.5231 (2)	0.0715 (8)	
C1	0.7242 (7)	0.1814 (5)	0.7699 (7)	0.054 (3)	
C2	0.9092 (8)	0.2425 (4)	0.4226 (7)	0.052 (2)	
C3	0.4656 (6)	0.3405 (4)	0.5286 (5)	0.040 (2)	
H3A	0.4440	0.3789	0.5097	0.048*	
H3B	0.4248	0.3128	0.4946	0.048*	
C4	0.5943 (7)	0.3337 (4)	0.3880 (6)	0.044 (2)	
C5	0.6844 (8)	0.3277 (4)	0.3492 (7)	0.059 (3)	
H5	0.7459	0.3256	0.3822	0.071*	
C6	0.6847 (8)	0.3246 (5)	0.2606 (7)	0.069 (3)	
H6	0.7465	0.3211	0.2346	0.083*	
C7	0.5942 (8)	0.3266 (5)	0.2106 (7)	0.066 (3)	
H7	0.5947	0.3244	0.1513	0.079*	
C8	0.5053 (8)	0.3316 (4)	0.2487 (6)	0.058 (3)	
H8	0.4438	0.3320	0.2156	0.069*	
C9	0.5049 (7)	0.3363 (4)	0.3366 (6)	0.049 (2)	
H9	0.4428	0.3413	0.3618	0.059*	
C10	0.6664 (7)	0.3976 (4)	0.5373 (6)	0.041 (2)	
C11	0.7642 (7)	0.3934 (4)	0.5762 (6)	0.056 (3)	
H11	0.7946	0.3572	0.5837	0.067*	
C12	0.8174 (8)	0.4423 (4)	0.6041 (7)	0.063 (3)	
H12	0.8831	0.4388	0.6300	0.076*	
C13	0.7733 (8)	0.4958 (4)	0.5936 (7)	0.061 (3)	
H13	0.8082	0.5287	0.6137	0.073*	
C14	0.6787 (8)	0.5008 (4)	0.5539 (7)	0.061 (3)	
H14	0.6494	0.5373	0.5460	0.073*	
C15	0.6250 (7)	0.4521 (4)	0.5250 (6)	0.051 (2)	
H15	0.5606	0.4562	0.4971	0.061*	
C16	0.2954 (7)	0.3366 (4)	0.6316 (6)	0.045 (2)	
C17	0.2480 (7)	0.3291 (4)	0.7076 (7)	0.058 (3)	
H17	0.2870	0.3247	0.7590	0.070*	
C18	0.1413 (8)	0.3280 (5)	0.7065 (8)	0.073 (3)	
H18	0.1086	0.3223	0.7571	0.088*	
C19	0.0856 (9)	0.3353 (5)	0.6317 (9)	0.072 (3)	
H19	0.0144	0.3352	0.6317	0.086*	
C20	0.1301 (8)	0.3428 (5)	0.5569 (8)	0.072 (3)	
H20	0.0902	0.3467	0.5060	0.086*	
C21	0.2356 (7)	0.3445 (4)	0.5569 (7)	0.060 (3)	
H21	0.2668	0.3510	0.5059	0.072*	
C22	0.4750 (7)	0.4014 (4)	0.6861 (6)	0.041 (2)	
C23	0.5642 (7)	0.4043 (4)	0.7378 (6)	0.051 (2)	
H23	0.5998	0.3706	0.7529	0.062*	
C24	0.6009 (8)	0.4577 (4)	0.7673 (7)	0.064 (3)	
H24	0.6614	0.4595	0.8017	0.077*	
C25	0.5495 (9)	0.5072 (5)	0.7463 (7)	0.063 (3)	
H25	0.5758	0.5428	0.7649	0.076*	
C26	0.4596 (8)	0.5049 (4)	0.6982 (6)	0.056 (3)	
H26	0.4232	0.5387	0.6856	0.067*	
C27	0.4220 (7)	0.4523 (4)	0.6680 (6)	0.048 (2)	
H27	0.3604	0.4510	0.6351	0.058*	
C28	0.5626 (7)	0.1018 (4)	0.6134 (6)	0.047 (2)	
H28A	0.6162	0.0938	0.6574	0.056*	
H28B	0.5372	0.0648	0.5914	0.056*	
C29	0.7291 (7)	0.0990 (4)	0.4987 (6)	0.044 (2)	
C30	0.8073 (7)	0.0897 (4)	0.5609 (7)	0.057 (3)	
H30	0.7958	0.0973	0.6180	0.068*	
C31	0.9021 (8)	0.0694 (5)	0.5398 (8)	0.069 (3)	
H31	0.9547	0.0648	0.5821	0.083*	
C32	0.9181 (9)	0.0561 (5)	0.4572 (8)	0.069 (3)	
H32	0.9812	0.0415	0.4429	0.082*	
C33	0.8420 (9)	0.0641 (5)	0.3955 (7)	0.069 (3)	
H33	0.8531	0.0546	0.3390	0.083*	
C34	0.7467 (8)	0.0864 (4)	0.4158 (7)	0.059 (3)	
H34	0.6956	0.0925	0.3728	0.071*	
C35	0.5264 (7)	0.1321 (4)	0.4371 (6)	0.048 (2)	
C36	0.4939 (8)	0.0789 (5)	0.4059 (7)	0.068 (3)	
H36	0.5227	0.0453	0.4296	0.081*	
C37	0.4185 (9)	0.0750 (5)	0.3393 (8)	0.087 (4)	
H37	0.3971	0.0387	0.3192	0.104*	
C38	0.3753 (8)	0.1241 (5)	0.3028 (7)	0.072 (3)	
H38	0.3228	0.1213	0.2601	0.087*	
C39	0.4105 (8)	0.1773 (5)	0.3301 (7)	0.064 (3)	
H39	0.3841	0.2108	0.3042	0.077*	
C40	0.4854 (7)	0.1809 (4)	0.3963 (6)	0.055 (3)	
H40	0.5089	0.2173	0.4141	0.066*	
C41	0.3476 (7)	0.1354 (4)	0.5868 (6)	0.050 (2)	
C42	0.3018 (8)	0.1858 (4)	0.5572 (6)	0.057 (3)	
H42	0.3296	0.2208	0.5765	0.069*	
C43	0.2166 (8)	0.1872 (5)	0.5002 (7)	0.068 (3)	
H43	0.1883	0.2225	0.4828	0.082*	
C44	0.1741 (8)	0.1366 (5)	0.4695 (8)	0.075 (3)	
H44	0.1136	0.1369	0.4349	0.090*	
C45	0.2227 (10)	0.0855 (6)	0.4910 (9)	0.093 (4)	
H45	0.1965	0.0505	0.4698	0.111*	
C46	0.3107 (9)	0.0863 (5)	0.5444 (8)	0.080 (4)	
H46	0.3474	0.0520	0.5522	0.096*	
C47	0.4310 (8)	0.0900 (4)	0.7463 (7)	0.058 (3)	
C48	0.4558 (10)	0.1033 (5)	0.8299 (8)	0.086 (4)	
H48	0.4851	0.1394	0.8419	0.103*	
C49	0.4401 (11)	0.0665 (6)	0.8987 (9)	0.101 (5)	
H49	0.4587	0.0786	0.9542	0.121*	
C50	0.3977 (10)	0.0128 (5)	0.8854 (8)	0.086 (4)	
H50	0.3875	−0.0122	0.9307	0.103*	
C51	0.3709 (12)	−0.0023 (6)	0.8029 (10)	0.108 (5)	
H51	0.3412	−0.0383	0.7908	0.130*	
C52	0.3882 (11)	0.0358 (5)	0.7370 (9)	0.095 (4)	
H52	0.3690	0.0237	0.6816	0.114*	

### Atomic displacement parameters (Å^2^)

**Table:**

	*U*^11^	*U*^22^	*U*^33^	*U*^12^	*U*^13^	*U*^23^
Ag1	0.0510 (4)	0.0350 (4)	0.0509 (5)	0.0026 (3)	−0.0054 (3)	0.0020 (3)
Ag2	0.0420 (4)	0.0364 (4)	0.0477 (4)	−0.0003 (3)	−0.0025 (3)	0.0017 (3)
N1	0.071 (7)	0.083 (8)	0.114 (9)	0.002 (6)	−0.006 (6)	0.033 (7)
N2	0.109 (8)	0.087 (7)	0.062 (7)	−0.013 (6)	0.052 (6)	−0.016 (5)
P1	0.0366 (12)	0.0327 (12)	0.0335 (13)	0.0025 (10)	0.0034 (10)	0.0027 (9)
P2	0.0357 (12)	0.0317 (12)	0.0360 (13)	0.0029 (10)	0.0056 (10)	0.0000 (9)
P3	0.0361 (12)	0.0326 (12)	0.0320 (13)	−0.0006 (10)	0.0018 (10)	0.0012 (9)
P4	0.0375 (12)	0.0330 (11)	0.0441 (14)	−0.0037 (10)	0.0097 (11)	−0.0011 (10)
S1	0.0394 (12)	0.0609 (16)	0.0453 (14)	−0.0077 (11)	−0.0038 (10)	−0.0030 (12)
S2	0.0556 (16)	0.094 (2)	0.0655 (19)	−0.0031 (16)	0.0120 (14)	−0.0011 (16)
C1	0.039 (5)	0.060 (7)	0.062 (7)	0.001 (5)	−0.002 (5)	0.010 (5)
C2	0.060 (6)	0.042 (6)	0.057 (7)	−0.003 (5)	0.012 (5)	−0.002 (5)
C3	0.044 (5)	0.038 (5)	0.038 (5)	−0.001 (4)	0.003 (4)	−0.002 (4)
C4	0.046 (5)	0.048 (6)	0.039 (5)	0.000 (4)	0.008 (4)	0.002 (4)
C5	0.054 (6)	0.077 (8)	0.047 (7)	−0.005 (5)	0.010 (5)	0.003 (5)
C6	0.058 (7)	0.101 (9)	0.051 (7)	−0.007 (6)	0.018 (6)	−0.004 (6)
C7	0.068 (7)	0.085 (8)	0.044 (7)	−0.004 (6)	0.009 (6)	0.001 (6)
C8	0.055 (6)	0.074 (7)	0.044 (6)	0.001 (5)	0.001 (5)	−0.002 (5)
C9	0.047 (6)	0.060 (6)	0.041 (6)	0.004 (5)	0.007 (5)	0.001 (5)
C10	0.050 (6)	0.036 (5)	0.038 (5)	−0.005 (4)	0.006 (4)	0.002 (4)
C11	0.058 (6)	0.049 (6)	0.059 (7)	−0.003 (5)	−0.008 (5)	0.000 (5)
C12	0.061 (7)	0.056 (7)	0.070 (8)	−0.014 (6)	−0.011 (6)	−0.002 (6)
C13	0.071 (8)	0.048 (7)	0.062 (7)	−0.014 (6)	0.001 (6)	−0.010 (5)
C14	0.074 (8)	0.042 (6)	0.067 (8)	−0.006 (5)	0.006 (6)	0.003 (5)
C15	0.055 (6)	0.043 (6)	0.055 (7)	−0.001 (5)	0.002 (5)	0.006 (5)
C16	0.038 (5)	0.041 (5)	0.058 (7)	0.002 (4)	0.005 (5)	0.001 (4)
C17	0.045 (6)	0.064 (7)	0.066 (7)	0.001 (5)	0.008 (5)	−0.005 (5)
C18	0.053 (7)	0.084 (9)	0.085 (10)	−0.001 (6)	0.021 (7)	−0.007 (7)
C19	0.045 (6)	0.078 (8)	0.092 (10)	0.005 (6)	0.007 (7)	−0.011 (7)
C20	0.048 (7)	0.080 (8)	0.085 (9)	0.010 (6)	−0.009 (6)	−0.005 (7)
C21	0.049 (6)	0.063 (7)	0.068 (8)	0.004 (5)	−0.001 (5)	−0.002 (6)
C22	0.045 (5)	0.038 (5)	0.042 (6)	0.001 (4)	0.006 (4)	0.001 (4)
C23	0.060 (6)	0.044 (6)	0.049 (6)	−0.002 (5)	−0.004 (5)	−0.001 (5)
C24	0.069 (7)	0.056 (7)	0.065 (8)	−0.007 (6)	−0.010 (6)	−0.009 (6)
C25	0.081 (8)	0.051 (7)	0.057 (7)	−0.014 (6)	0.008 (6)	−0.006 (5)
C26	0.077 (8)	0.038 (6)	0.053 (7)	0.003 (5)	0.008 (6)	−0.001 (5)
C27	0.057 (6)	0.042 (6)	0.046 (6)	0.002 (5)	0.003 (5)	−0.001 (4)
C28	0.050 (6)	0.043 (5)	0.048 (6)	−0.003 (4)	0.003 (5)	0.003 (4)
C29	0.045 (5)	0.047 (6)	0.041 (6)	0.004 (4)	0.006 (5)	−0.002 (4)
C30	0.051 (6)	0.064 (7)	0.055 (7)	0.011 (5)	0.005 (5)	−0.007 (5)
C31	0.055 (7)	0.080 (8)	0.073 (9)	0.011 (6)	0.002 (6)	−0.010 (6)
C32	0.059 (7)	0.075 (8)	0.073 (9)	0.012 (6)	0.014 (6)	0.001 (6)
C33	0.077 (8)	0.074 (8)	0.059 (8)	0.016 (6)	0.020 (6)	−0.007 (6)
C34	0.060 (7)	0.065 (7)	0.052 (7)	0.015 (5)	0.005 (5)	0.004 (5)
C35	0.046 (5)	0.046 (6)	0.049 (6)	0.000 (4)	−0.008 (5)	0.003 (4)
C36	0.072 (7)	0.059 (7)	0.069 (8)	−0.005 (6)	−0.025 (6)	0.009 (6)
C37	0.089 (9)	0.076 (9)	0.089 (10)	−0.013 (7)	−0.035 (8)	0.004 (7)
C38	0.061 (7)	0.081 (9)	0.071 (8)	−0.002 (6)	−0.022 (6)	0.011 (7)
C39	0.060 (7)	0.072 (8)	0.059 (7)	0.016 (6)	−0.013 (6)	0.010 (6)
C40	0.052 (6)	0.057 (7)	0.054 (7)	0.007 (5)	−0.006 (5)	0.006 (5)
C41	0.046 (6)	0.048 (6)	0.055 (6)	−0.006 (5)	−0.003 (5)	−0.001 (5)
C42	0.061 (7)	0.049 (6)	0.060 (7)	−0.004 (5)	−0.010 (5)	−0.002 (5)
C43	0.065 (7)	0.068 (8)	0.070 (8)	0.002 (6)	−0.014 (6)	0.000 (6)
C44	0.058 (7)	0.076 (9)	0.087 (9)	0.000 (6)	−0.021 (6)	−0.004 (7)
C45	0.083 (9)	0.080 (9)	0.110 (11)	−0.014 (7)	−0.033 (8)	−0.005 (8)
C46	0.081 (9)	0.065 (8)	0.090 (10)	−0.004 (6)	−0.022 (7)	0.000 (7)
C47	0.075 (7)	0.045 (6)	0.055 (7)	−0.015 (5)	0.016 (6)	−0.001 (5)
C48	0.114 (11)	0.071 (8)	0.070 (9)	−0.033 (7)	−0.012 (8)	0.020 (7)
C49	0.143 (13)	0.088 (10)	0.071 (9)	−0.031 (9)	−0.012 (9)	0.023 (7)
C50	0.115 (11)	0.073 (9)	0.070 (9)	−0.022 (8)	0.009 (8)	0.024 (7)
C51	0.158 (15)	0.080 (10)	0.089 (11)	−0.050 (9)	0.024 (10)	0.015 (8)
C52	0.134 (12)	0.076 (9)	0.076 (9)	−0.034 (8)	0.013 (9)	0.006 (7)

### Geometric parameters (Å, °)

**Table:**

Ag1—P1	2.450 (2)	C21—H21	0.9300
Ag1—P3	2.451 (2)	C22—C23	1.379 (12)
Ag1—S1	2.670 (3)	C22—C27	1.384 (12)
Ag1—S2	2.768 (3)	C23—C24	1.392 (13)
Ag2—N2^i^	2.429 (9)	C23—H23	0.9300
Ag2—P2	2.497 (2)	C24—C25	1.355 (14)
Ag2—P4	2.498 (2)	C24—H24	0.9300
Ag2—S1	2.668 (2)	C25—C26	1.357 (14)
N1—C1	1.142 (12)	C25—H25	0.9300
N2—C2	1.153 (12)	C26—C27	1.382 (12)
N2—Ag2^ii^	2.429 (9)	C26—H26	0.9300
P1—C4	1.817 (9)	C27—H27	0.9300
P1—C10	1.819 (9)	C28—H28A	0.9700
P1—C3	1.842 (8)	C28—H28B	0.9700
P2—C22	1.815 (9)	C29—C34	1.361 (13)
P2—C3	1.821 (9)	C29—C30	1.384 (12)
P2—C16	1.825 (9)	C30—C31	1.385 (13)
P3—C35	1.797 (9)	C30—H30	0.9300
P3—C29	1.819 (9)	C31—C32	1.355 (14)
P3—C28	1.838 (9)	C31—H31	0.9300
P4—C47	1.783 (10)	C32—C33	1.356 (14)
P4—C41	1.806 (9)	C32—H32	0.9300
P4—C28	1.855 (9)	C33—C34	1.402 (13)
S1—C1	1.659 (11)	C33—H33	0.9300
S2—C2	1.633 (11)	C34—H34	0.9300
C3—H3A	0.9700	C35—C36	1.379 (13)
C3—H3B	0.9700	C35—C40	1.386 (12)
C4—C5	1.364 (13)	C36—C37	1.393 (14)
C4—C9	1.380 (12)	C36—H36	0.9300
C5—C6	1.387 (13)	C37—C38	1.375 (15)
C5—H5	0.9300	C37—H37	0.9300
C6—C7	1.378 (14)	C38—C39	1.370 (14)
C6—H6	0.9300	C38—H38	0.9300
C7—C8	1.342 (13)	C39—C40	1.384 (13)
C7—H7	0.9300	C39—H39	0.9300
C8—C9	1.378 (13)	C40—H40	0.9300
C8—H8	0.9300	C41—C42	1.375 (13)
C9—H9	0.9300	C41—C46	1.385 (14)
C10—C15	1.378 (12)	C42—C43	1.383 (13)
C10—C11	1.386 (12)	C42—H42	0.9300
C11—C12	1.382 (13)	C43—C44	1.367 (14)
C11—H11	0.9300	C43—H43	0.9300
C12—C13	1.369 (13)	C44—C45	1.373 (15)
C12—H12	0.9300	C44—H44	0.9300
C13—C14	1.355 (13)	C45—C46	1.379 (15)
C13—H13	0.9300	C45—H45	0.9300
C14—C15	1.388 (13)	C46—H46	0.9300
C14—H14	0.9300	C47—C48	1.363 (15)
C15—H15	0.9300	C47—C52	1.374 (14)
C16—C21	1.377 (13)	C48—C49	1.396 (15)
C16—C17	1.383 (13)	C48—H48	0.9300
C17—C18	1.394 (13)	C49—C50	1.368 (16)
C17—H17	0.9300	C49—H49	0.9300
C18—C19	1.351 (15)	C50—C51	1.362 (17)
C18—H18	0.9300	C50—H50	0.9300
C19—C20	1.348 (15)	C51—C52	1.383 (16)
C19—H19	0.9300	C51—H51	0.9300
C20—C21	1.380 (14)	C52—H52	0.9300
C20—H20	0.9300		
			
P1—Ag1—P3	127.18 (8)	C16—C21—C20	120.8 (11)
P1—Ag1—S1	108.67 (8)	C16—C21—H21	119.6
P3—Ag1—S1	106.10 (8)	C20—C21—H21	119.6
P1—Ag1—S2	107.71 (9)	C23—C22—C27	118.3 (8)
P3—Ag1—S2	104.55 (9)	C23—C22—P2	119.2 (7)
S1—Ag1—S2	99.11 (8)	C27—C22—P2	122.4 (7)
N2^i^—Ag2—P2	101.4 (2)	C22—C23—C24	119.9 (9)
N2^i^—Ag2—P4	113.2 (2)	C22—C23—H23	120.0
P2—Ag2—P4	126.43 (8)	C24—C23—H23	120.0
N2^i^—Ag2—S1	101.5 (3)	C25—C24—C23	120.7 (10)
P2—Ag2—S1	106.66 (8)	C25—C24—H24	119.7
P4—Ag2—S1	104.95 (8)	C23—C24—H24	119.7
C2—N2—Ag2^ii^	163.2 (10)	C24—C25—C26	120.1 (10)
C4—P1—C10	104.3 (4)	C24—C25—H25	120.0
C4—P1—C3	102.5 (4)	C26—C25—H25	120.0
C10—P1—C3	106.5 (4)	C25—C26—C27	120.1 (10)
C4—P1—Ag1	111.9 (3)	C25—C26—H26	119.9
C10—P1—Ag1	116.5 (3)	C27—C26—H26	119.9
C3—P1—Ag1	113.9 (3)	C26—C27—C22	120.8 (9)
C22—P2—C3	102.3 (4)	C26—C27—H27	119.6
C22—P2—C16	104.1 (4)	C22—C27—H27	119.6
C3—P2—C16	101.6 (4)	P3—C28—P4	114.6 (5)
C22—P2—Ag2	119.0 (3)	P3—C28—H28A	108.6
C3—P2—Ag2	116.4 (3)	P4—C28—H28A	108.6
C16—P2—Ag2	111.3 (3)	P3—C28—H28B	108.6
C35—P3—C29	105.0 (4)	P4—C28—H28B	108.6
C35—P3—C28	103.6 (4)	H28A—C28—H28B	107.6
C29—P3—C28	105.2 (4)	C34—C29—C30	118.4 (9)
C35—P3—Ag1	117.6 (3)	C34—C29—P3	121.4 (7)
C29—P3—Ag1	108.1 (3)	C30—C29—P3	118.8 (7)
C28—P3—Ag1	116.1 (3)	C29—C30—C31	121.2 (10)
C47—P4—C41	105.6 (5)	C29—C30—H30	119.4
C47—P4—C28	100.1 (4)	C31—C30—H30	119.4
C41—P4—C28	105.8 (4)	C32—C31—C30	119.7 (11)
C47—P4—Ag2	114.9 (3)	C32—C31—H31	120.1
C41—P4—Ag2	113.3 (3)	C30—C31—H31	120.1
C28—P4—Ag2	115.7 (3)	C31—C32—C33	119.8 (11)
C1—S1—Ag2	99.6 (3)	C31—C32—H32	120.1
C1—S1—Ag1	109.4 (4)	C33—C32—H32	120.1
Ag2—S1—Ag1	81.47 (7)	C32—C33—C34	120.8 (11)
C2—S2—Ag1	117.9 (4)	C32—C33—H33	119.6
N1—C1—S1	179.3 (12)	C34—C33—H33	119.6
N2—C2—S2	165.5 (11)	C29—C34—C33	119.9 (10)
P2—C3—P1	116.9 (4)	C29—C34—H34	120.1
P2—C3—H3A	108.1	C33—C34—H34	120.1
P1—C3—H3A	108.1	C36—C35—C40	117.3 (9)
P2—C3—H3B	108.1	C36—C35—P3	124.0 (7)
P1—C3—H3B	108.1	C40—C35—P3	118.7 (7)
H3A—C3—H3B	107.3	C35—C36—C37	120.8 (10)
C5—C4—C9	118.0 (9)	C35—C36—H36	119.6
C5—C4—P1	117.0 (7)	C37—C36—H36	119.6
C9—C4—P1	124.8 (7)	C38—C37—C36	120.7 (11)
C4—C5—C6	120.2 (10)	C38—C37—H37	119.6
C4—C5—H5	119.9	C36—C37—H37	119.6
C6—C5—H5	119.9	C39—C38—C37	119.2 (10)
C7—C6—C5	120.7 (10)	C39—C38—H38	120.4
C7—C6—H6	119.6	C37—C38—H38	120.4
C5—C6—H6	119.6	C38—C39—C40	119.8 (10)
C8—C7—C6	119.2 (10)	C38—C39—H39	120.1
C8—C7—H7	120.4	C40—C39—H39	120.1
C6—C7—H7	120.4	C39—C40—C35	122.1 (10)
C7—C8—C9	120.4 (10)	C39—C40—H40	118.9
C7—C8—H8	119.8	C35—C40—H40	118.9
C9—C8—H8	119.8	C42—C41—C46	113.5 (9)
C8—C9—C4	121.5 (9)	C42—C41—P4	118.9 (7)
C8—C9—H9	119.3	C46—C41—P4	127.0 (8)
C4—C9—H9	119.3	C41—C42—C43	123.6 (10)
C15—C10—C11	117.9 (8)	C41—C42—H42	118.2
C15—C10—P1	123.8 (7)	C43—C42—H42	118.2
C11—C10—P1	118.2 (7)	C44—C43—C42	120.0 (10)
C12—C11—C10	121.0 (9)	C44—C43—H43	120.0
C12—C11—H11	119.5	C42—C43—H43	120.0
C10—C11—H11	119.5	C43—C44—C45	118.4 (10)
C13—C12—C11	119.9 (10)	C43—C44—H44	120.8
C13—C12—H12	120.0	C45—C44—H44	120.8
C11—C12—H12	120.0	C44—C45—C46	119.6 (11)
C14—C13—C12	119.9 (9)	C44—C45—H45	120.2
C14—C13—H13	120.0	C46—C45—H45	120.2
C12—C13—H13	120.0	C45—C46—C41	123.6 (11)
C13—C14—C15	120.6 (10)	C45—C46—H46	118.2
C13—C14—H14	119.7	C41—C46—H46	118.2
C15—C14—H14	119.7	C48—C47—C52	112.3 (10)
C10—C15—C14	120.6 (9)	C48—C47—P4	120.4 (8)
C10—C15—H15	119.7	C52—C47—P4	127.3 (9)
C14—C15—H15	119.7	C47—C48—C49	124.3 (12)
C21—C16—C17	119.0 (9)	C47—C48—H48	117.9
C21—C16—P2	125.6 (8)	C49—C48—H48	117.9
C17—C16—P2	115.4 (7)	C50—C49—C48	120.8 (13)
C16—C17—C18	119.4 (10)	C50—C49—H49	119.6
C16—C17—H17	120.3	C48—C49—H49	119.6
C18—C17—H17	120.3	C51—C50—C49	117.1 (12)
C19—C18—C17	119.7 (11)	C51—C50—H50	121.5
C19—C18—H18	120.2	C49—C50—H50	121.5
C17—C18—H18	120.2	C50—C51—C52	119.8 (13)
C20—C19—C18	121.9 (11)	C50—C51—H51	120.1
C20—C19—H19	119.0	C52—C51—H51	120.1
C18—C19—H19	119.0	C47—C52—C51	125.8 (13)
C19—C20—C21	119.2 (11)	C47—C52—H52	117.1
C19—C20—H20	120.4	C51—C52—H52	117.1
C21—C20—H20	120.4		
			
P3—Ag1—P1—C4	−57.1 (3)	C3—P2—C16—C21	−0.2 (9)
S1—Ag1—P1—C4	174.4 (3)	Ag2—P2—C16—C21	−124.8 (8)
S2—Ag1—P1—C4	67.9 (3)	C22—P2—C16—C17	−76.7 (8)
P3—Ag1—P1—C10	−176.9 (3)	C3—P2—C16—C17	177.2 (7)
S1—Ag1—P1—C10	54.5 (3)	Ag2—P2—C16—C17	52.7 (8)
S2—Ag1—P1—C10	−52.0 (3)	C21—C16—C17—C18	1.7 (15)
P3—Ag1—P1—C3	58.6 (3)	P2—C16—C17—C18	−175.9 (8)
S1—Ag1—P1—C3	−70.0 (3)	C16—C17—C18—C19	−1.1 (16)
S2—Ag1—P1—C3	−176.5 (3)	C17—C18—C19—C20	1.1 (18)
N2^i^—Ag2—P2—C22	51.2 (4)	C18—C19—C20—C21	−1.7 (18)
P4—Ag2—P2—C22	−178.4 (3)	C17—C16—C21—C20	−2.3 (15)
S1—Ag2—P2—C22	−54.6 (3)	P2—C16—C21—C20	175.0 (8)
N2^i^—Ag2—P2—C3	174.4 (4)	C19—C20—C21—C16	2.3 (16)
P4—Ag2—P2—C3	−55.1 (3)	C3—P2—C22—C23	−105.2 (8)
S1—Ag2—P2—C3	68.6 (3)	C16—P2—C22—C23	149.2 (8)
N2^i^—Ag2—P2—C16	−69.8 (4)	Ag2—P2—C22—C23	24.7 (9)
P4—Ag2—P2—C16	60.7 (4)	C3—P2—C22—C27	71.4 (8)
S1—Ag2—P2—C16	−175.6 (3)	C16—P2—C22—C27	−34.1 (9)
P1—Ag1—P3—C35	13.1 (4)	Ag2—P2—C22—C27	−158.7 (7)
S1—Ag1—P3—C35	142.7 (4)	C27—C22—C23—C24	−2.6 (14)
S2—Ag1—P3—C35	−113.1 (4)	P2—C22—C23—C24	174.2 (8)
P1—Ag1—P3—C29	131.7 (3)	C22—C23—C24—C25	0.5 (16)
S1—Ag1—P3—C29	−98.8 (3)	C23—C24—C25—C26	2.0 (17)
S2—Ag1—P3—C29	5.4 (3)	C24—C25—C26—C27	−2.2 (16)
P1—Ag1—P3—C28	−110.5 (3)	C25—C26—C27—C22	0.0 (15)
S1—Ag1—P3—C28	19.1 (3)	C23—C22—C27—C26	2.4 (14)
S2—Ag1—P3—C28	123.3 (3)	P2—C22—C27—C26	−174.3 (7)
N2^i^—Ag2—P4—C47	−14.5 (5)	C35—P3—C28—P4	−77.0 (6)
P2—Ag2—P4—C47	−140.2 (4)	C29—P3—C28—P4	173.0 (5)
S1—Ag2—P4—C47	95.3 (4)	Ag1—P3—C28—P4	53.5 (5)
N2^i^—Ag2—P4—C41	107.1 (5)	C47—P4—C28—P3	−176.6 (5)
P2—Ag2—P4—C41	−18.7 (4)	C41—P4—C28—P3	73.9 (6)
S1—Ag2—P4—C41	−143.2 (4)	Ag2—P4—C28—P3	−52.5 (6)
N2^i^—Ag2—P4—C28	−130.4 (4)	C35—P3—C29—C34	24.1 (9)
P2—Ag2—P4—C28	103.8 (3)	C28—P3—C29—C34	133.1 (8)
S1—Ag2—P4—C28	−20.7 (3)	Ag1—P3—C29—C34	−102.2 (8)
N2^i^—Ag2—S1—C1	79.4 (5)	C35—P3—C29—C30	−169.4 (8)
P2—Ag2—S1—C1	−174.8 (4)	C28—P3—C29—C30	−60.5 (9)
P4—Ag2—S1—C1	−38.6 (4)	Ag1—P3—C29—C30	64.2 (8)
N2^i^—Ag2—S1—Ag1	−172.2 (2)	C34—C29—C30—C31	1.6 (15)
P2—Ag2—S1—Ag1	−66.44 (8)	P3—C29—C30—C31	−165.3 (8)
P4—Ag2—S1—Ag1	69.74 (8)	C29—C30—C31—C32	−2.6 (17)
P1—Ag1—S1—C1	166.8 (4)	C30—C31—C32—C33	1.6 (18)
P3—Ag1—S1—C1	27.2 (4)	C31—C32—C33—C34	0.5 (18)
S2—Ag1—S1—C1	−80.9 (4)	C30—C29—C34—C33	0.5 (15)
P1—Ag1—S1—Ag2	69.48 (8)	P3—C29—C34—C33	167.0 (8)
P3—Ag1—S1—Ag2	−70.11 (8)	C32—C33—C34—C29	−1.5 (17)
S2—Ag1—S1—Ag2	−178.23 (8)	C29—P3—C35—C36	53.5 (10)
P1—Ag1—S2—C2	−62.2 (4)	C28—P3—C35—C36	−56.6 (10)
P3—Ag1—S2—C2	75.4 (4)	Ag1—P3—C35—C36	173.7 (8)
S1—Ag1—S2—C2	−175.2 (4)	C29—P3—C35—C40	−126.1 (8)
Ag2—S1—C1—N1	119 (96)	C28—P3—C35—C40	123.9 (8)
Ag1—S1—C1—N1	35 (96)	Ag1—P3—C35—C40	−5.8 (9)
Ag2^ii^—N2—C2—S2	−149 (2)	C40—C35—C36—C37	−3.5 (17)
Ag1—S2—C2—N2	−169 (4)	P3—C35—C36—C37	176.9 (9)
C22—P2—C3—P1	78.9 (5)	C35—C36—C37—C38	0(2)
C16—P2—C3—P1	−173.7 (5)	C36—C37—C38—C39	3(2)
Ag2—P2—C3—P1	−52.6 (5)	C37—C38—C39—C40	−2.9 (18)
C4—P1—C3—P2	173.2 (5)	C38—C39—C40—C35	−0.3 (17)
C10—P1—C3—P2	−77.6 (6)	C36—C35—C40—C39	3.5 (16)
Ag1—P1—C3—P2	52.2 (5)	P3—C35—C40—C39	−176.9 (8)
C10—P1—C4—C5	69.3 (8)	C47—P4—C41—C42	132.2 (9)
C3—P1—C4—C5	−179.9 (8)	C28—P4—C41—C42	−122.3 (8)
Ag1—P1—C4—C5	−57.5 (8)	Ag2—P4—C41—C42	5.5 (10)
C10—P1—C4—C9	−116.6 (8)	C47—P4—C41—C46	−57.0 (11)
C3—P1—C4—C9	−5.7 (9)	C28—P4—C41—C46	48.6 (11)
Ag1—P1—C4—C9	116.6 (8)	Ag2—P4—C41—C46	176.4 (9)
C9—C4—C5—C6	0.2 (15)	C46—C41—C42—C43	9.5 (16)
P1—C4—C5—C6	174.8 (8)	P4—C41—C42—C43	−178.4 (9)
C4—C5—C6—C7	−1.1 (17)	C41—C42—C43—C44	−0.7 (18)
C5—C6—C7—C8	0.1 (18)	C42—C43—C44—C45	−5.3 (19)
C6—C7—C8—C9	1.7 (17)	C43—C44—C45—C46	2(2)
C7—C8—C9—C4	−2.5 (16)	C44—C45—C46—C41	8(2)
C5—C4—C9—C8	1.5 (14)	C42—C41—C46—C45	−13.3 (18)
P1—C4—C9—C8	−172.6 (8)	P4—C41—C46—C45	175.4 (10)
C4—P1—C10—C15	67.9 (9)	C41—P4—C47—C48	−140.9 (10)
C3—P1—C10—C15	−40.0 (9)	C28—P4—C47—C48	109.4 (11)
Ag1—P1—C10—C15	−168.2 (7)	Ag2—P4—C47—C48	−15.2 (12)
C4—P1—C10—C11	−111.3 (8)	C41—P4—C47—C52	40.1 (13)
C3—P1—C10—C11	140.8 (7)	C28—P4—C47—C52	−69.6 (12)
Ag1—P1—C10—C11	12.6 (9)	Ag2—P4—C47—C52	165.7 (10)
C15—C10—C11—C12	1.9 (15)	C52—C47—C48—C49	0(2)
P1—C10—C11—C12	−178.8 (8)	P4—C47—C48—C49	−179.0 (11)
C10—C11—C12—C13	0.1 (16)	C47—C48—C49—C50	0(2)
C11—C12—C13—C14	−1.7 (17)	C48—C49—C50—C51	−1(2)
C12—C13—C14—C15	1.1 (17)	C49—C50—C51—C52	1(2)
C11—C10—C15—C14	−2.5 (14)	C48—C47—C52—C51	0(2)
P1—C10—C15—C14	178.3 (8)	P4—C47—C52—C51	178.9 (12)
C13—C14—C15—C10	1.0 (16)	C50—C51—C52—C47	0(3)
C22—P2—C16—C21	105.8 (9)		

Symmetry codes: (i) *x*−1/2, −*y*+1/2, *z*+1/2; (ii) *x*+1/2, −*y*+1/2, *z*−1/2.
